# Combined numerical and experimental biomechanical characterization of soft collagen hydrogel substrate

**DOI:** 10.1007/s10856-016-5688-3

**Published:** 2016-02-25

**Authors:** A. P. G. Castro, P. Laity, M. Shariatzadeh, C. Wittkowske, C. Holland, D. Lacroix

**Affiliations:** Department of Mechanical Engineering, INSIGNEO Institute for in Silico Medicine, The University of Sheffield, Pam Liversidge Building - Room F32, Mappin Street, Sheffield, S1 3JD UK; Natural Materials Group, Department of Materials Science and Engineering, The University of Sheffield, Sheffield, UK

## Abstract

This work presents a combined experimental–numerical framework for the biomechanical characterization of highly hydrated collagen hydrogels, namely with 0.20, 0.30 and 0.40 % (by weight) of collagen concentration. Collagen is the most abundant protein in the extracellular matrix of animals and humans. Its intrinsic biocompatibility makes collagen a promising substrate for embedding cells within a highly hydrated environment mimicking natural soft tissues. Cell behaviour is greatly influenced by the mechanical properties of the surrounding matrix, but the biomechanical characterization of collagen hydrogels has been challenging up to now, since they present non-linear poro-viscoelastic properties. Combining the stiffness outcomes from rheological experiments with relevant literature data on collagen permeability, poroelastic finite element (FE) models were developed. Comparison between experimental confined compression tests available in the literature and analogous FE stress relaxation curves showed a close agreement throughout the tests. This framework allowed establishing that the dynamic shear modulus of the collagen hydrogels is between 0.0097 ± 0.018 kPa for the 0.20 % concentration and 0.0601 ± 0.044 kPa for the 0.40 % concentration. The Poisson’s ratio values for such conditions lie within the range of 0.495–0.485 for 0.20 % and 0.480–0.470 for 0.40 %, respectively, showing that rheology is sensitive enough to detect these small changes in collagen concentration and thus allowing to link rheology results with the confined compression tests. In conclusion, this integrated approach allows for accurate constitutive modelling of collagen hydrogels. This framework sets the grounds for the characterization of related hydrogels and to the use of this collagen parameterization in more complex multiscale models.

## Introduction

Collagen-based hydrogels have a wide range of tissue engineering (TE) applications, given the importance and abundance of this structural protein in organic systems. Collagen is one of the major components of extracellular matrix, existent in approximately 30 % of all musculoskeletal tissues. It is biocompatible and presents low immunogenicity, being mostly used as a cell substrate, by providing an advantageous environment for tissue growth and organ regeneration. It is also used in the production of scaffolds or scaffold’s coatings [1, 2, 6, 12, 32].

There is a need for the biomechanical characterization of collagen, either as a hydrogel component or as isolated fibres, since most of its biological functions are intrinsically associated with the biomechanics of the tissues [27]. As collagen is present at different levels, it is essential to consider a multiscale approach, from the cellular interaction to the organ level. The contribution of the fibrils is also determinant for the overall biomechanics of the construct or application [20, 23, 24].

Collagen is known to present non-linear poro-viscoelastic properties, which means that its characterization is challenging, both experimentally and numerically [18]. The properties of collagen depend particularly on its origin, concentration, crosslinking and sample preparation techniques. Stiffness properties or hydraulic permeability of collagen hydrogels were experimentally measured by several groups [5, 14, 29], and studied numerically [26, 31]. In addition, having in mind that direct determination of soft tissues Poisson’s ratio is a highly demanding task, the discussion on the definition of this parameter pointed out that the overall domain of most soft tissues is almost incompressible, being particularly sensitive to the properties of the soft matrix [3, 13, 17]. However, the overall approach to biological hydrogels tends to adopt a Poisson’s ratio between 0.20 and 0.30 for the solid part [14].

Rheology is one of the most commonly used techniques for the evaluation of biomechanical properties of soft tissues and hydrogels. Several groups have tested different types of collagen hydrogels using this method, in order to obtain information on the shear and viscous behaviour of such materials. The dynamic nature of rheological experiments mostly allows for the definition of the hydrogel’s solid phase, since the fluid is not able to instantaneously move through the porous solid [10, 13, 14].

On one hand, Velegol and Lanni [29] aimed at determining the local shear modulus of type I collagen. Their samples were hydrogels with 0.05–0.23 % concentration of bovine dermal collagen. Frequency sweeps between 0.06 and 60 rad/s were performed at 22 and 37 °C in a parallel plate rheometer. The shear modulus of these hydrogels was determined in the range of 3–80 Pa.

On the other hand, Wu et al. [30] focused on finding the age-related differences in rat-tail collagen over a frequency sweep from 0.1 to 25 rad/s. The experiments were run at 25 °C, using a cone plate support with 2° angle and 60 mm diameter. They evidenced a direct relationship between stiffness and ageing.

However, Knapp et al. [14] affirmed that rheology is not able to account for the interphase drag of biphasic materials, being restricted to a monophasic characterization. Therefore, this group combined rheometer experiments with confined compression tests, which allow for fluid exudation. This integrated approach allowed them to conclude that the microstructural organization of the fibrils plays an essential role on collagen behaviour, by influencing both tension and compression responses, at the solid–fluid interaction level. However, this work also pointed out that creep data over compression experiments is hardly linear, being particularly influenced by the compression rate. This means that high compression rates (“step”) generate fast fluid exudation, which is hardly measurable. Chandran and Barocas [9] and Busby et al. [5] also performed confined compression with collagen hydrogels. While Busby et al. [5] focused on steady-pace compression rate experiments (“ramp”), Chandran and Barocas [9] performed both “step” and “ramp”-like compression rates. Such experiments are one of the most reliable approaches for the determination of the biphasic behaviour of this type of hydrogels, but both groups faced several difficulties in obtaining a stable creep deformation.

Numerical simulations can provide significant accuracy in analogous tests, thus overcoming these experimental issues, particularly in what concerns to “ramp” confined compression tests. Therefore, this work presents an experimental and numerical framework for the biomechanical characterization of highly hydrated collagen hydrogels, namely with 0.20, 0.30 and 0.40 % (by weight) of collagen concentration.

The experimental characterization through rheology is based on a protocol similar to the one applied by Velegol and Lanni [29], aiming to determine the stiffness properties of the hydrogels under dynamic conditions.

The additional biphasic characterization through FE confined compression tests follows the protocols and testing configurations of Chandran and Barocas [9] and Busby et al. [5]. The numerical outcomes are compared with the analogous experimental results, in order to define a set of biomechanical parameters that can describe the behaviour of each hydrogel.

The present work is part of a wider multiscale biomechanical framework that is studying the macroscopic load transfer from a scaffold to the local microscopic stimuli at the cell level, as found in many TE applications. Dynamic compressive strains were shown to influence cell fate, so one needs to characterize the mechanics of the microenvironment [11]. Collagen is a common choice for such microenvironment constructs [16, 25]. Therefore, this study aims to contribute to the understanding of how collagen behaves mechanically as a scaffold template for cells to attach in TE applications so that such outcome can later be used in biomechanical and mechanobiological studies investigating the local mechanical stimuli at the cell level [6, 19, 32].

## Materials and methods

### Specimen preparation

Type I bovine collagen (LifeTechnologies, USA) was neutralized by adding 10× phosphate-buffered saline solution (PBS), 1 N NaOH and distilled water. Four collagen hydrogel samples were made at each of the three different collagen concentrations (0.20, 0.30 and 0.40 % by weight, or 2, 3 and 4 mg/ml, respectively). The specimens (20 mm diameter, 1 mm height) were prepared by pipetting approximately 400 μl of the hydrogel mixture on marked areas of parafilm. Incubation of the hydrogel at 37 °C in a humidified incubator for 40 min initiated the gelling of the gels. Finally, the samples were released from the parafilm and kept hydrated in PBS. One sample for each concentration of 0.20 and 0.40 % were damaged when handling between the sample holder and the rheometer, and were therefore discarded.

### Rheological experiments

Rheological characterisation was performed using a Bohlin Gemini 200 rheometer (Malvern Instruments, Malvern, United Kingdom), fitted with a 20 mm diameter (PP20) flat plate geometry and a Peltier heating stage, which controlled the specimen temperature at 37 °C. Hydrogel specimens with different collagen concentrations, namely 0.20, 0.30 and 0.40 % by weight, were carefully placed on the rheometer stage and the geometry was closed to a gap of 850 µm, which compressed the specimen very slightly (by ca. 15 %). The area around the specimen was flooded with PBS/distilled water and covered with an environmental cuff to prevent it drying out. The experimental protocol was based on a frequency sweep from 3 to 0.012 Hz (descending order), on a total of 15 frequency steps, using an oscillatory strain of 0.01. The average duration for a full frequency sweep was 10 min. However, equipment limitations on testing such a soft material only allowed reliable measurements to be obtained under certain frequencies, depending on the hydrogel concentration, i.e., under 0.70 Hz for 0.20 %, under 1.40 Hz for 0.30 % and 2.10 Hz for 0.40 %. Consequently, measurements at higher frequencies were excluded from each experimental dataset.

The dynamic (or complex) shear modulus ($G^{*}$) is related to the elastic (or storage, $G^{\prime }$) and viscous (or loss, $G^{\prime \prime}$) moduli as follows:

1 $$G{^{*}}\left( \omega \right) = G^{\prime}\left( \omega \right) + iG^{\prime\prime}\left( \omega \right)$$

Subsequently, the magnitude of $G^{*}$ can be obtained through Eq. 2:

2 $$\left| {G^{*}\left( \omega \right)} \right| = \sqrt {G^{{\prime 2}} \left( \omega \right) + G^{{\prime \prime 2}} \left( \omega \right)}$$

Hence, for the three groups of samples, plots of moduli versus frequency (*ω*) were obtained from the oscillatory measurements. The calculated average dynamic shear modulus served as input for the numerical simulations.

### Finite element simulations

The biomechanical behaviour of collagen hydrogels was modelled through FE simulations with the biphasic module of V-Biomech [7, 8]. For validation purposes, the simulations replicated the protocols of the confined compression experiments of Chandran and Barocas [9] and Busby et al. [5], respectively. Chandran and Barocas [9] used rectangular parallelepiped hydrogel samples of 3 × 15 × 15 mm, with 0.30 % of collagen concentration (bovine origin). A cuboidal model with the same dimensions was meshed with 14,161 nodes, using 1536 quadratic 27-node hexahedral elements (b)

**Fig. 1:**
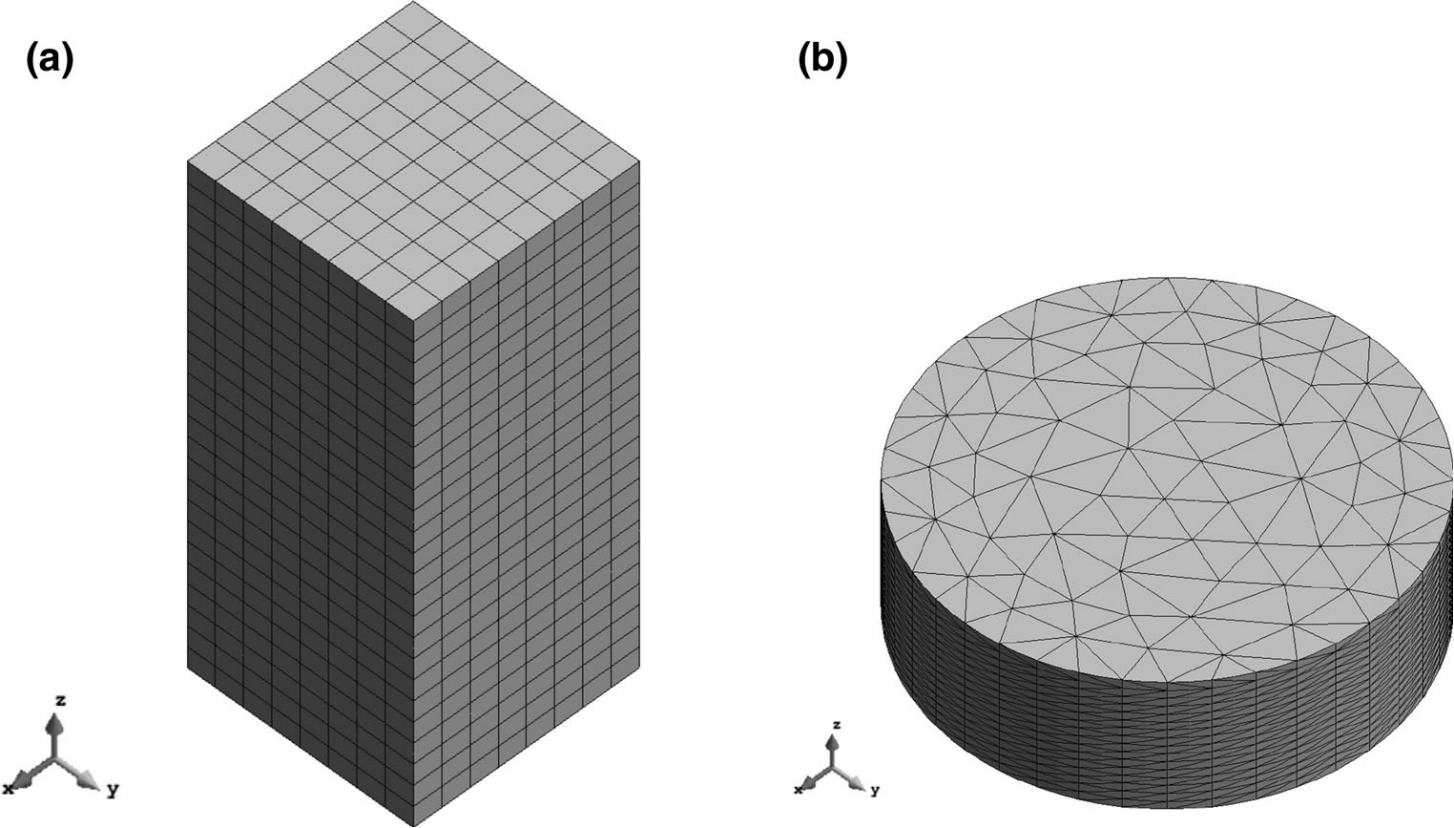
FE models: **a** rectangular sample of Chandran and Barocas [9]; **b** cylindrical sample of Busby et al. [5]

Figure 1a. The protocol was divided in compression and relaxation stages: (1) 10 % compression at 0.001 s^−1^ and, (2) compression hold for 2100 s (stress relaxation period).

Busby et al. [5] used cylindrical samples of radius 8 mm and height 5 mm, considering hydrogels with 0.20, 0.30 and 0.40 % of collagen concentration (rat-tail origin). A cylindrical model with the same dimensions was meshed with 11,385 nodes, using 7296 quadratic 10-node tetrahedral elements (Fig. 1b). The protocol was also divided in compression and relaxation stages: (1) 5 % compression at 0.005 s^−1^ and, (2) compression hold for 300 s (stress relaxation period).

The boundary conditions for both cases considered bottom and lateral confinement (X- and Y-axis), with the compression applied at the top (Z-axis). Fluid exudation was allowed through the top.

A nonlinear poroelastic approach was followed to model the nonlinear behaviour of the hydrogels. No viscoelasticity was considered for these particular trials, due to the long-term compression rates applied [21]. A compressible Neo-Hookean model [4] was used to characterize the isotropic hyperelasticity of the collagen hydrogel, in terms of shear ($G$) and bulk moduli ($K$):

3 $$\overline{W}_{\text{NH}} \left( {\mathbf{C}} \right) = \frac{G}{2}\left( {I_{1} - 3} \right) - G(lnJ) - \frac{G}{3}(lnJ)^{2} + \frac{K}{2}(lnJ)^{2}$$

In addition, the van der Voet model was applied to describe the strain-dependent permeability, $K_{{}}^{*}$ [8, 27, 28]:

4 $$K_{{}}^{*} \left( J \right) = K_{0}^{*} J^{M}$$

where $I_{1}$ is the first invariant,$J$ is the determinant of the transformation gradient, $K_{0}^{*}$ is the zero-strain hydraulic permeability and $M$ is a dimensionless nonlinear permeability parameter. These parameters, provided by Busby et al, are shown in Table 1.

**Table 1:** Constitutive parameters of the collagen hydrogels

Collagen concentration (%)	$Ha$ (kPa)	$K_{0}^{*}$ (m^4^/Ns)	$M$
0.20	0.90	1.70 × 10^−10^	1.8
0.30	1.00	1.20 × 10^−10^	2.1
0.40	1.20	0.80 × 10^−10^	3.5

Adapted from Busby et al. [5]

Equation 5 expresses the conversion from aggregate modulus ($Ha$) to Young’s modulus ($E$), as a function of Poisson’s ratio ($\nu$). Equations 6 and 7 lead to the determination of shear and bulk moduli [15, 22]. It must be highlighted that the later measures of the compressibility of the material.

5 $$Ha = E\frac{{\left( {1 - \nu } \right)}}{{\left( {1 + \nu } \right)\left( {1 - 2\nu } \right)}}$$

6 $$G = \frac{E}{{2\left( {1 + \nu } \right)}}$$

7 $$K = \frac{E}{{3\left( {1 - 2\nu } \right)}}$$

The numerical outputs were longitudinal stress ($\sigma_{zz}$) versus time plots for to each case. Different Poisson’s ratio are selected, in accordance with rheology results. Overall, the simulations comprised four confined compression tests with the rectangular parallelepiped FE model and 0.30 % of collagen concentration [9] and twelve confined compression tests with the cylindrical FE model, corresponding to the three different collagen concentrations used by Busby et al. [5]. By simulating two different setups, which were performed with collagen from different origins, one aims to establish and validate an independent set of parameters for highly hydrated collagen hydrogels.

## Results

### Rheological experiments

**Fig. 2:**
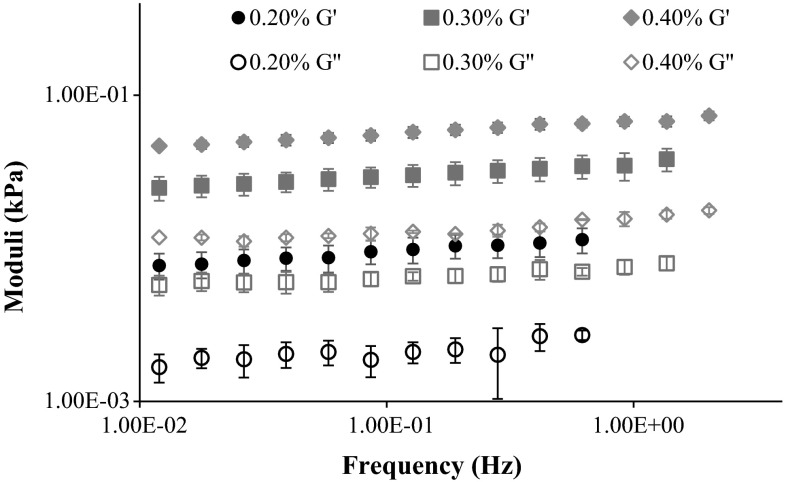
Average and standard deviations of elastic and viscous moduli as a function of the frequency for the three different collagen concentration levels

Figure 2 shows the average values of elastic and viscous moduli for the three collagen concentration levels. For the 0.20 % hydrogel, the elastic modulus varied from 0.0146 kPa at 1.3 Hz to 0.0077 kPa at 0.012 Hz, while the viscous modulus varied between 0.0037 kPa and 0.0017 kPa. Average standard deviations were 0.0019 and 0.0005 kPa, respectively. For the 0.30 % hydrogel, the elastic modulus varied from 0.0382 kPa at 1.3 Hz to 0.0249 kPa at 0.012 Hz, while the viscous modulus varied between 0.0080 kPa and 0.0058 kPa. Average standard deviations were 0.0051 kPa and 0.0008 kPa, respectively. Finally, for the 0.40 % hydrogel, the elastic modulus varied from 0.0675 kPa at 1.3 Hz to 0.0468 kPa at 0.012 Hz, while the viscous modulus varied between 0.0116 and 0.0118 kPa. Average standard deviations were 0.0044 and 0.0007 kPa, respectively.

**Fig. 3:**
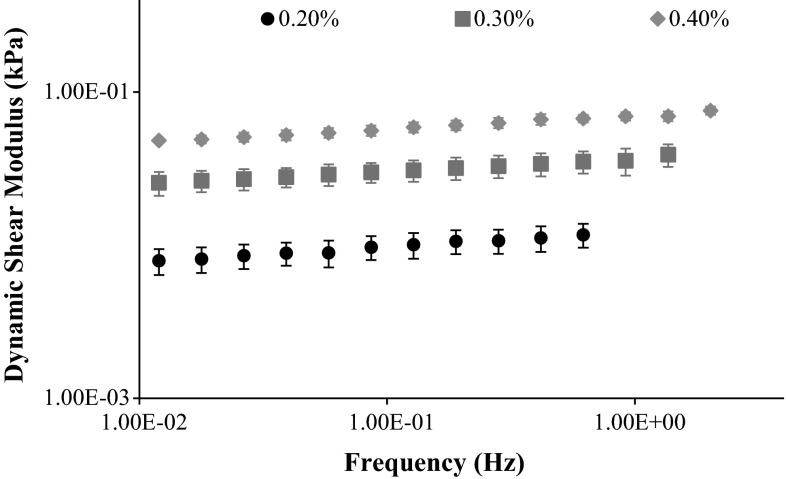
Calculated dynamic shear modulus of the three different hydrogels, as a function of the frequency

The configurations of both moduli versus frequency curves were very similar for each level of collagen concentration. The viscous moduli were on average 20 % of the corresponding elastic modulus. Figure 3 illustrates the comparison between the behaviour of the different hydrogels, in terms of dynamic shear modulus.

Table 2 summarizes the average values for dynamic shear, elastic and viscous moduli as functions of collagen concentration, along with the corresponding average standard deviation. The samples with 0.40 % of collagen displayed the lowest average standard deviation, while the 0.20 % ones presented the highest.

**Table 2:** Average values for dynamic shear, elastic and viscous moduli as a function of collagen concentration, with the corresponding average standard deviation

Collagen concentration (%)	$G$ (kPa)		$G^{\prime }$ (kPa)		$G^{\prime \prime }$ (kPa)	
	Average	Standard deviation	Average	Standard deviation	Average	Standard deviation
0.20	0.0097	0.0018	0.0094	0.0017	0.0021	0.0004
0.30	0.0311	0.0052	0.0304	0.0051	0.0066	0.0008
0.40	0.0601	0.0044	0.0585	0.0045	0.0134	0.0008

### Finite element simulations

Table 3 shows shear and bulk moduli values applied on the numerical simulations, calculated as a function of the (unknown) Poisson’s ratio and the aggregate modulus ($Ha$) provided by Busby et al. [5]. Knapp et al. [14] stated that collagen hydrogels should have a Poisson’s ratio between 0.20 and 0.30, but higher Poisson’s ratios show closer correspondence with the experimental characterization.

**Table 3:** Shear and bulk moduli calculated as function of the Poisson’s ratio

Collagen concentration (%)	$\nu$	$Ha$ (kPa)	$G$ (kPa)	$K$ (kPa)
0.20	0.200	0.90	0.3375	0.4500
	0.300		0.2571	0.5571
	0.485		0.0262	0.8650
	0.495		0.0089	0.8881
0.30	0.200	1.00	0.3750	0.5000
	0.300		0.2857	0.6190
	0.480		0.0385	0.9487
	0.490		0.0196	0.9739
0.40	0.200	1.20	0.4500	0.6000
	0.300		0.3429	0.7429
	0.470		0.0679	1.1094
	0.480		0.0462	1.1385

Aggregate modulus extracted from the work of Busby et al. [5]

**Fig. 4:**
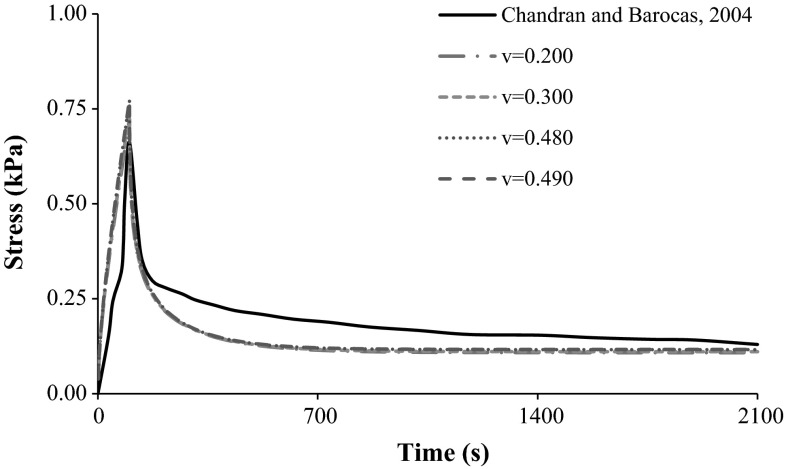
Stress-time curve of 0.30 % collagen hydrogel during 10 % compression at 0.001 s^−1^ and 2100 s relaxation. Comparison between the experimental data of Chandran and Barocas [9] and the numerical calculations with different Poisson’s ratio values

Figure 4 and Table 4 show the comparison between the experimental stress curve of Chandran and Barocas [9] and the related numerical calculations (longitudinal stress). Four different Poisson’s ratio values were considered, namely 0.200, 0.300, 0.480 and 0.490, in accordance with the rheology results for the hydrogel with 0.30 % of collagen concentration (average dynamic shear modulus and standard deviation) and Table 3.

**Table 4:** Comparison between the experimental results of Chandran and Barocas [9] and the FE calculations, considering peak and relaxation stress values

Collagen concentration (%)	Case	Peak stress at 100 s (kPa)	Relaxation stress at 2100 s (kPa)
0.30	Chandran and Barocas [9]	0.662	0.130
	ν = 0.200	0.724	0.108
	ν = 0.300	0.738	0.110
	ν = 0.480	0.771	0.116
	ν = 0.490	0.773	0.117

Table 4 shows that the peak and relaxation stress values from the FE calculations are significantly close to those reported by Chandran and Barocas [9]. Peak stress values were on average 14 % higher than the experimental results (between 9 % for ν = 0.200 and 17 % for ν = 0.490), whereas the stress values at the end of the test were on average 12 % lower. Figure 4 shows that the mechanical behaviour of collagen during the experimental tests was fairly reproduced by the numerical models for most of test’s duration, even if those have predicted a faster stress relaxation.

**Fig. 5:**
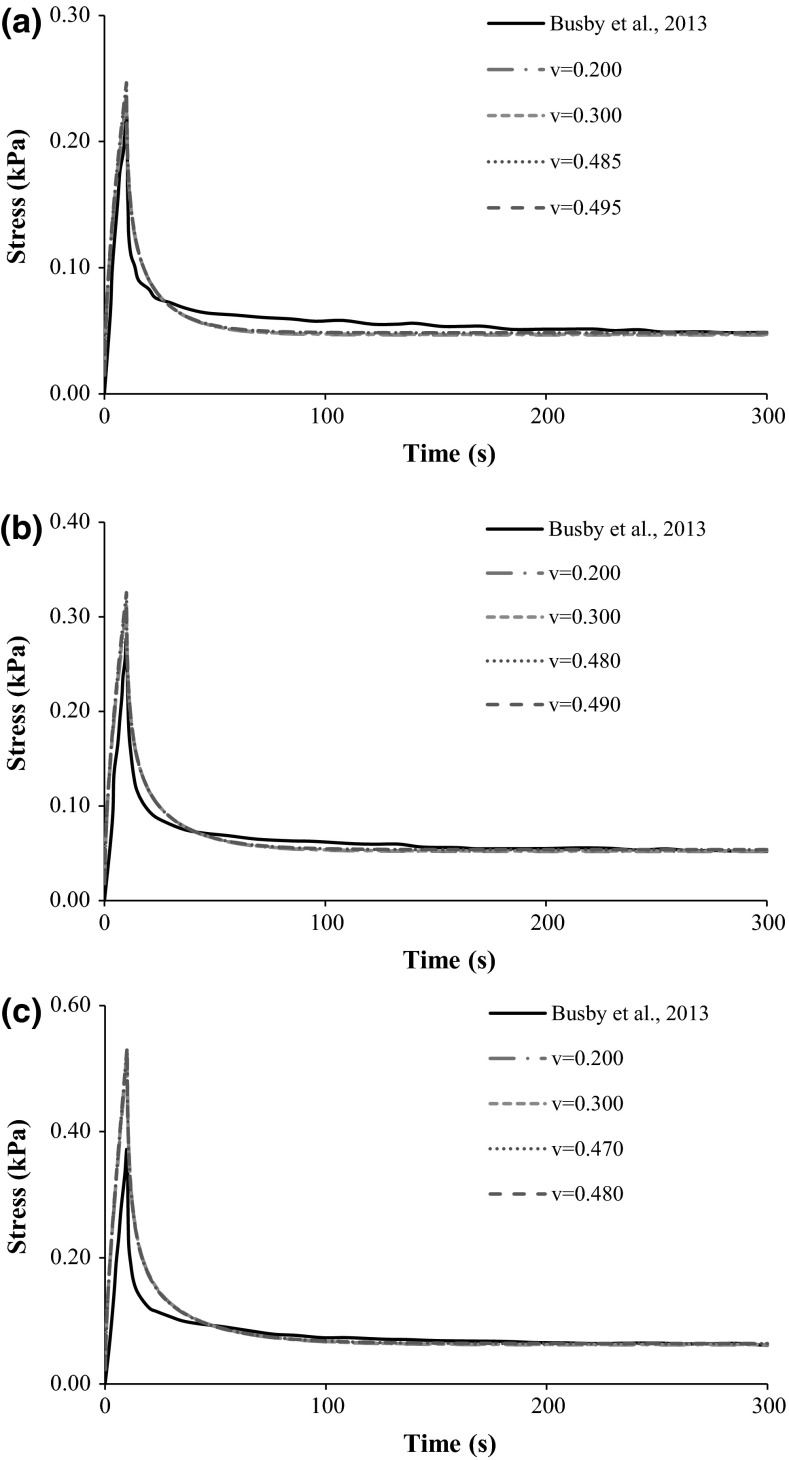
Stress-time curves of collagen hydrogels during 5 % compression at 0.005 s^−1^ and 300 s relaxation. Comparison between the experimental data of Busby et al. [5] and the numerical calculations with different Poisson’s ratio values, in respect to different collagen concentration levels. **a** 0.20 %; **b** 0.30 %; **c** 0.40 %

Figure 5 and Table 5 show the comparison between the experimental stress curves of Busby et al. [5] and the analogous numerical calculations (longitudinal stress). Following the procedure applied to the previous simulations, four different Poisson’s ratio values were considered for each collagen concentration level. Having that 0.200 and 0.300 were replicated in each level, the other values were the following: (1) 0.485 and 0.490 for 0.20 %, (2) 0.480 and 0.490 for 0.30 %, and (3) 0.470 and 0.480 for 0.40 %. These specific values were selected in accordance with the rheology results for each hydrogel, considering the average dynamic shear modulus values and the respective standard deviation, as stated in Table 3.

**Table 5:** Comparison between the experimental results of Busby et al. [5] and the FE calculations, considering peak and relaxation stress values

Collagen concentration (%)	Case	Peak stress at 10 s (kPa)	Relaxation stress at 300 s (kPa)
0.20	Busby et al. [5]	0.223	0.049
	ν = 0.200	0.235	0.047
	ν = 0.300	0.238	0.047
	ν = 0.485	0.246	0.048
	ν = 0.495	0.247	0.049
0.30	Busby et al. [5]	0.275	0.052
	ν = 0.200	0.310	0.052
	ν = 0.300	0.315	0.052
	ν = 0.480	0.325	0.054
	ν = 0.490	0.326	0.054
0.40	Busby et al. [5]	0.369	0.061
	ν = 0.200	0.523	0.062
	ν = 0.300	0.530	0.063
	ν = 0.470	0.532	0.064
	ν = 0.480	0.533	0.065

Table 5 demonstrates that the variations on the Poisson’s ratio were not significant for the three groups of numerical simulations (four simulations per group), as the models of each group produced similar peak and relaxation stress values. For the 0.20 % group, peak stress values were on average 9 % higher than the experimental results (between 5 % for ν = 0.200 and 11 % for ν = 0.495), and the relaxation stress values were almost identical (less than 2 % average difference). For the 0.30 % group, peak stress values were on average 17 % higher than the experimental results (between 13 % for ν = 0.200 and 18 % for ν = 0.490), while the relaxation stress values were less than 3 % higher (average). Finally, for the 0.40 % group, peak stress values were approximately 44 % higher than the experimental results (between 42 % for ν = 0.200 and 44 % for ν = 0.480), while the relaxation stress values were approximately 4 % higher.

Figure 5 shows that the numerical stress versus time curves were positively close to the corresponding experimental curves. The differences in the relaxation stage observed in the previous case are here much less significant.

## Discussion

In this study, a combined experimental and numerical approach was used to test the mechanical behaviour of collagen hydrogels. The results of the rheometer experiments are within the range of several literature studies on collagen rheology [14, 20, 29–31], as both elastic and viscous moduli decreased slowly with decreased frequency for the three different levels of collagen concentration. The viscous modulus of each hydrogel group was approximately 20 % of the corresponding elastic modulus, in line with the work of Knapp et al. [14]. The simple experimental procedure (10 min testing time for each hydrogel sample and no need for complex sample mounting on the equipment) is a good compromise with the quality of the abovementioned results. Rheological experiments also proved to be sensitive enough to detect the small increases on the collagen concentration of the hydrogels, as the Poisson’s ratio laid between 0.495 for 0.20 % and 0.470 for 0.40 %.

Nevertheless, rheology is only able to provide relevant stiffness data, so FE simulations were essential to provide substantial data on the multiphasic stress relaxation behaviour of these hydrogels. The link between the stiffness properties extracted from the rheometer experiments and the parameters provided by Busby et al. [5] resulted in a significant agreement between the numerical models and the experimental studies of Chandran and Barocas [9] and Busby et al. [5]. An overall tendency for higher stress peak values in the numerical models was registered, which is probably related to the fluid exudation conditions that were fully unconstrained in the numerical model. Differences in the peak stress were greater at higher collagen concentrations, which may have been caused by the increasing rigidity of the hydrogel. The 0.20 % models registered on average 9 % higher peak stresses, while the 0.40 % models differed on average 44 %, in comparison to the experiment data by Busby et al. [5]. However, much smaller differences were found at the end of the relaxation test, with differences lower than 2–4 %, respectively. As theoretically expected, increases in the Poisson’s ratio corresponded to slightly higher stress values, all along the test.

A similar qualitative and quantitative behaviour was registered for the data generated with the FE models with 0.30 % of collagen concentration and the two experimental tests with analogous hydrogels. The average differences were 14 and 12 % higher than the experiment of Chandran and Barocas [9] and 17 and 3 % higher in respect to Busby et al. [5], for the peak stress and stress relaxation stages, respectively.

These results prove that the constitutive modelling applied in the FE simulations is independent from the configuration of the model. This means that the outcomes of the FE simulations are only associated with the material properties of the hydrogels, rather than being influenced by the type of element, the dimensions of the model or the applied strain rate.

The stress curves calculated by the FE models followed the corresponding experimental outcomes, which reinforces the reliability and applicability of the numerical modelling for further studies involving collagen in a multiscale framework. Nevertheless, the experimental curve obtained by Chandran and Barocas [9] showed a gradual stress decrease, which was to some extent different from the more immediate stress decrease recorded by Busby et al. [5] and calculated by the sixteen FE models, even if the peak and relaxation stress values were as close as previously mentioned (less than 15 % average difference). This slower stress stabilization of Chandran and Barocas samples may be related to the experimental conditions, namely with friction on the compressive plates, enhanced by the rectangular shape of the compression chamber. The numerical simulations did not consider any friction effects, which were also not observed on the work of Busby et al. [5]. It is very likely that cylindrical compression chambers allow faster fluid exudation and lower friction.

The approach proposed in this study should be analysed in the light of different assumptions and limitations. No permeability-related experiments were performed, which means that the work presented by Busby et al. [5] was the primary source of data on aggregate modulus, zero-strain hydraulic permeability and nonlinear permeability coefficient of collagen hydrogels. However, the comparison of the results of this study with the results from Chandran and Barocas [9] showed that the behaviour of the numerical models are valid under a wide range of conditions. In fact, Busby et al. [5] used rat-tail collagen, which could have conditioned the comparison with the bovine collagen used on the rheological experiments and also on the work of Chandran and Barocas [9], but the origin difference turned out as not significant. Cross-linking and sample preparation approaches were also negligible, but they could have played a role if this study was further extended on the biological level.

In what concerns to the discussion on the Poisson’s ratio, Knapp et al. [14] pointed out that collagen hydrogels should be compressible, as previously mentioned. This reference indicates values of Poisson’s ratio ranging between 0.20 and 0.30. However, if one assumes that the Poisson’s ratio of the overall domain of this collagen hydrogel is almost incompressible (higher than 0.45), the aggregate modulus values extracted from the work of Busby et al. match the experimental stiffness properties from the rheometer, for the different concentration levels. This behaviour was expected, due to rheology’s dynamic nature [10, 14]. The assumption of incompressibility is essential to link the experimental rheology data with the data from confined compression tests.

This work confirms that assuming the overall domain of the collagen hydrogels as almost incompressible is adequate for highly dynamic conditions [3, 10, 13], at the same time that the stiffness-related link between rheology and confined compression tests demonstrates that Poisson’s ratio is lower in less dynamic settings. The inverse engineering approach implemented in this work showed to be a viable strategy to combine inputs from different sources, in order to define a comprehensive set of parameters that describe the biomechanical behaviour of these hydrogels.

## Conclusion

Rheology is a reliable method to determine the mechanical properties of soft tissues. The short duration of the experiments and the absence of specific testing jigs are also attractive, particularly in comparison with confined compression experiments. Using this technique, the stiffness properties of hydrogels with different collagen concentrations (0.20, 0.30 and 0.40 %) were successfully characterized and validated in this work, regardless of the minor differences amongst them.

The versatility and increasing speed of in silico FE studies allow to overcome intrinsic experimental difficulties, if one has access to reliable data. The stiffness results from the rheometer experiments were combined with relevant literature data on collagen’s permeability to develop an accurate collagen’s nonlinear poroelastic FE model. The comparison between numerical and experimental stress relaxation curves [5, 9] showed a close agreement in both peak and relaxation stress.

To sum up, the current study is a relevant step forward on the constitutive modelling of collagen hydrogels, due to its integrated experimental–numerical approach, which may now be applied to the characterization of related hydrogels. Given the importance of collagen for TE applications and its determinant role as cell substrate, an accurate biomechanical characterization of this material is also important for the biomechanics and mechanobiology community.
